# Social Relationship Prediction Integrating Personality Traits and Asymmetric Interactions

**DOI:** 10.3389/fpsyg.2022.778722

**Published:** 2022-03-21

**Authors:** Chunhua Ju, Geyao Li, Fuguang Bao, Ting Gao, Yiling Zhu

**Affiliations:** ^1^Modern Business Research Center, Zhejiang Gongshang University, Hangzhou, China; ^2^School of Management and E-Business, Zhejiang Gongshang University, Hangzhou, China; ^3^Academy of Zhejiang Culture Industry Innovation & Development, Zhejiang Gongshang University, Hangzhou, China; ^4^School of Foreign Languages, Zhejiang Gongshang University, Hangzhou, China

**Keywords:** social network, personality traits, asymmetric interaction, ego-network, social relationship prediction

## Abstract

Social networks have become an important way for users to find friends and expand their social circle. Social networks can improve users’ experience by recommending more suitable friends to them. The key lies in improving the accuracy of link prediction, which is also the main research issue of this study. In the study of personality traits, some scholars have proved that personality can be used to predict users’ behavior in social networks. Based on these studies, this study aims to improve the accuracy of link prediction in directed social networks. Considering the integration of personality link preference and asymmetric interaction into the link prediction model of social networks, a four-dimensional link prediction model is proposed. Through comparative experiments, it is proved that the four-dimensional social relationship prediction model proposed in this study is more accurate than the model only based on similarity. At the same time, it is also verified that the matching degree of personality link preference and asymmetric interaction intensity in the model can help improve the accuracy of link prediction.

## Introduction

With the rapid development of mobile Internet, social networks have become an important way for people to find friends and expand their social circle. Nowadays, for many social network platforms, improving the accuracy of social relationship prediction is the key to success for it is helpful to enhance users’ experience, analyze consumers’ behavior, improve the public opinion environment, and recommend more suitable friends.

Online social network (OSN), with its rapidity, extensiveness, equality, and self-organization, develops in a short time and has a large number of users, which penetrates people’s lives. Link prediction is an important part of the social networks research. The solution to this problem plays a vital role in explaining the reasons for the formation of a network structure, helping us explore the law of networks evolution (Li et al., 2019) and understanding the mechanism of complex network (Li et al., 2015). In addition, link prediction can work to find friends in social networks, recommend projects in user-project websites, and find experts in academic networks (Xie et al., 2015). This paper, taking Sina Weibo (one of the most popular social network sites in China, just like Twitter overseas) as an example, focuses on the study of link prediction in social networks, attempting to discover and predict missing or possible relationships in the user relationship network.

With more and more attention paid to the research of link prediction, researchers in the fields of library, information science, communication science, sociology and computer science have put forward various solutions (Hu et al., 2017; Ju et al., 2022), some of which is mainly based on classification, some on probability graph model (PGM) and some on matrix factorization (MF). These methods have their own advantages from different perspectives. However, Wang et al. (2015), select representative pieces of literature and conducting classified statistics, found that researchers from different disciplines conducted studies on link prediction without cooperation. This study tries to break the discipline boundary and further explores the research of link prediction.

In social networks, individual differences are related to users’ feelings, attitudes, and reactions in communication and interaction, and ultimately, to social behaviors. However, personality, as a definition of individual difference, does not get enough attention in the pieces of social network research. The essence of link prediction is to estimate the possibility of link relationships between unlinked nodes based on the observation of link relationships, node attributes, and network structure attributes. It can also be understood as calculating the link preference of a node to other nodes, which is particularly effective when links are created between nodes (users) in a social network. At the same time, the personality theory believes that the user’s personality has a great influence on one’s preferences, and it has been proved that personality can be predicted through the social network of digital footprints. Therefore, this study aims to explore the relationship between personality and link preference in social networks by combining personality theory and link prediction. Considering the directional relationship between users on platforms such as Weibo, this study also considers the impact of asymmetric interaction on link prediction when constructing the social network connection prediction model. This study predicts users’ personalities by analyzing the data of social platforms and explores whether there is a connection between users with different personalities. Then, it comprehensively takes into consideration the users’ static attributes, network structure, the link preference of personality, and asymmetric interactions, and puts forward new four-dimensional link prediction models (FDLPM) whose effects are verified by experiments.

## Related Works

### Link Prediction

Link prediction is regarded as a basic problem of the social network’s evolution in time by Liben-Nowell and Kleinberg (2007), and they have proposed some classical prediction methods based on network topology information. It is common to measure the possibility of link generation by calculating the similarity between nodes since people usually establish new relationships with people who have certain similarities with them in topological or non-topological features (Bhattacharyya et al., 2011).

Topology-based measurement is defined by using various topological information of the network. Indicators, such as Common Neighbors (Lorrain et al., 1971) and Jaccard Coefficient (Jaccard, 1912), are generated by defining neighbor nodes as neighbors, which can indirectly reflect users’ social behaviors and directly affect users’ choices. Besides, there are other indicators. For example, Hu Ma et al. (2019) calculated the number of all paths between two nodes, and Friend Link (Chen et al., 2016) considered the path with length of L between nodes, which are all measuring indexes based on paths between nodes. There are also some measurements based on random walks, including Hitting Time (Fouss et al., 2007), an asymmetric measurement of the expected number of steps required for a random walk between nodes, as well as Prop Flow (Lichtenwalter et al., 2010), which is a more localized measurement.

Non-topological measurement focuses on information outside the network structure, such as the profile of users in social networks, including age, interests, geographic location and so on. Aiello et al. (2012) found that users’ tags could reflect their interests, so they finally proposed a method for link prediction based on tag similarity. In addition to the topological and non-topological measurements described above, link prediction can be viewed as a binary classification problem, where each pair of nodes is an instance, and positive and negative category labels indicate whether the node pair is connected. Many classification models have been applied to link prediction, such as the support vector machine (SVM) (Li and Chen, 2013) and k-nearest neighbor algorithm (KNN) (Zhu et al., 2017). Classification methods can also be considered as learning-based methods, the most critical part of which is the selection of features. The common neighbors or paths between two nodes can construct topological features, and a large number of experiments have proved that these topological features are effective in link prediction (Chiang et al., 2011). Also, it can construct non-topological features to improve the link prediction (Scellato et al., 2016). Moradabadi and Meybodi (2018) proposed a strategy of learning automata for link prediction in weighted social networks. Aziz et al. (2020) proposed a novel link prediction method that aims at improving the accuracy of existing path-based methods by incorporating information about the nodes along local paths. Tang R. et al. (2021) proposed a framework based on multiple types of consistency between embedding vectors (MulCEVs). In MulCEV, the traditional embedding-based method is applied to obtain the degree of consistency between the vectors representing the unmatched nodes, and a proposed distance consistency index based on the positions of nodes in each latent space provides additional clues for prediction. Mo et al. (2022) proposed a deep learning framework for temporal network link prediction. Bao et al. (2022) proposed an improved evaluation methodology for association rules and link prediction. Wei et al. (2022) proposed a novel time series-based graph model with text, called text with time series for graph (TT-Graph) model, which explicitly considers the user similarity and time series similarity. Link prediction applications, namely recommendation system, anomaly detection, influence analysis, and community detection become more strenuous due to network diversity and complex and dynamic network contexts (Daud et al., 2020).

To sum up, topological information between nodes in a network is the key to topological measurement and the topological feature-based learning model, and the validity of non-topological measurements and non-topological features depends on the external available information of their domain and specific network. As for Weibo, however, its large number of users and complicated user relationships may cause the problem of the lack of information when accessing network structure data. In order to protect privacy, non-topological information such as users’ profiles is incomplete. All these factors will directly affect the above methods, so it is the trend of current research to analyze potential features based on existing information. This study is trying to add more potential supplementary factors to the link prediction model.

### Personality Prediction

Personality traits are defined as endogenous, stable, hierarchical, and are influenced by biological factors such as genes and brain structure (Romero et al., 2009). The most commonly used model to describe personality is the Five Factor Model proposed by Goldberg (1990) and Costa and McCrae (1992). It holds the idea that personality is mainly determined by physiology and consists of five basic tendencies: openness to experience, extraversion, agreeableness, conscientiousness, and neuroticism. These traits are relatively stable throughout a person’s life cycle and under different situations, which is the reason why users’ personality traits can serve as a starting point for predicting users’ behavior. Ngai et al. (2015) emphasized that personality characteristics are generally considered as one of the basic theories to explain the influence of users’ subsequent behavior characteristics.

In recent years, scholars have begun to focus on the connection between personality and online social network behavior. Studies have shown that personality can be used to predict many aspects of life, including academic achievement (Komarraju et al., 2009), job performance (Neal et al., 2012), health status (Soldz and Vaillant, 1999), and social network behavior (Wang, 2013). McElroy et al. (2007) tested the general influence of personality on Internet use, and the results supported that personality should be taken as an explanatory factor. The big five personality traits could explain some of the differences in Internet use. Some scholars have preliminarily outlined a personalization-based approach. Hu and Pu (2011) attempted to solve the cold start problem by integrating collaborative filtering methods with personality traits. The so-called cold start problem refers to the dilemma of having no basic information to recommend (Ju et al., 2015).

In earlier studies, user’s personalities were obtained through questionnaires. In recent years, it has been proved that the big five personality traits are significantly correlated with behaviors in social networks. For example, people with high extroversion are more active in social networks and have more friends (Blackwell et al., 2017), while people with high neuroticism tend to hide themselves, try to understand others in a passive way, and use more negative words in their published content (Liu et al., 2016). Based on the above correlation, some scholars have tried to extract the personality traits of users from social networks directly. Kosinski et al. (2013) proved that users’ private attributes including personality traits could be predicted by digital records of users’ behaviors in online social networks, and they also proved the correlation between Facebook likes and personality traits.

Based on the above, it is concluded that the link between personality and social behavior has been demonstrated and personality can be predicted from social data. According to the Report on the Development of Weibo Users in 2020^1^, the number of daily active users of Weibo has reached 224 million, and a large amount of user-generated content like blog posts and interactive data is created every day. All of these are important unstructured information but without being fully used. This study aims to explore users’ personality potential characteristics from the data, and then make link prediction based on topological and non-topological features and personality traits of the network.

## Link Prediction Model Incorporating Personality Traits and Asymmetric Interactions

### Problem Description

**FIGURE 1:**
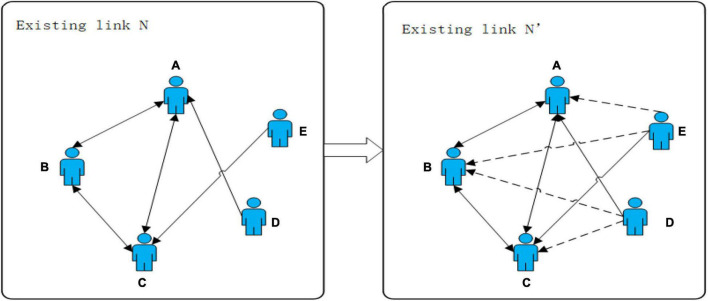
A sample diagram of a link prediction problem.

The link prediction mainly refers to predicting unknown links by using known social network information combined with personality traits. As shown in Figure 1, N is the known link (solid line), and N′ is the possible link (dashed line) predicted by calculating users’ similarity. This paper tries to solve the problem of predicting the result of N′ by using the known information in the existing link N. The closer the prediction result is to the real situation, the better it will be.

A social network is defined as a directed network *G*(*V*,*E*). *V* is the collection of nodes, and *E* is a collection of links. It is not allowed to duplicate links and self-links, *U* represents all possible sets of links, andU contains $\frac{\left|V\right|\cdot \left(\left|V\right|-1\right)}{2}$ links with |*V*|, representing the number of elements in set *V*, and *U-E* represents a collection of links that do not exist now. Assuming that there are some missing or coming links, then they are the target links, which the link prediction aims to find.

### Model Framework

**FIGURE 2:**
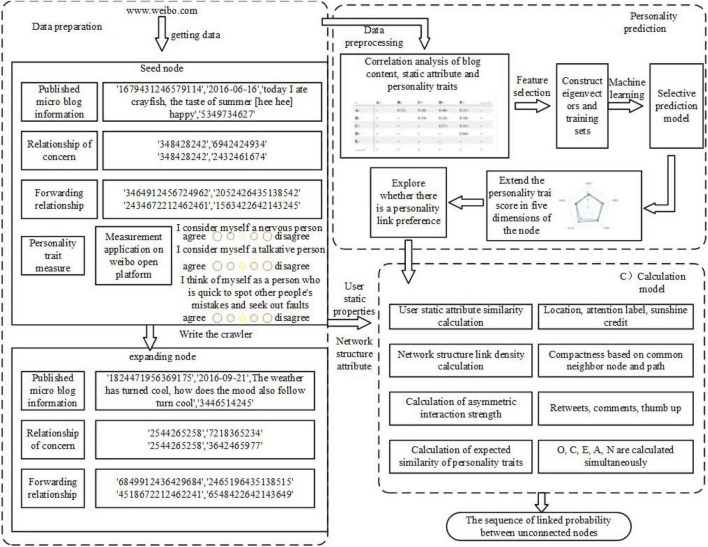
A link prediction model framework of four-dimensional social network.

Existing studies calculate the similarity between nodes based on existing link relations, node attributes, and network structure attributes so as to speculate missing links and possible link relations, but they all ignore the internal factors of personality traits. Combined with psychological research, this paper puts forward a method for social network link prediction integrating personality traits, and takes one of the most influential social media in China, Weibo, as an example. The FDLPM proposed in this paper is mainly composed of such three parts as data acquisition, personality traits prediction, and four-dimensional link prediction, as shown in Figure 2.

First of all, the authors developed an application for measuring the five personalities based on the Weibo platform. The content of the application is a recognized measuring table of the big five personality traits, with 44 tests of five dimensions of personality traits, which can reflect the performance of users’ personality traits in different dimensions. By inviting Weibo users to fill in a questionnaire, seed nodes are determined and five personality dimensions of the subjects are scored, respectively. Then, a web crawler program is written to obtain the blog content and behavior characteristics of users of the extension node. These characteristics have a follower and follower relationship with seed nodes. Secondly, taking seed nodes, blog content, behavior characteristics, and personality traits scores as learning samples, it uses machine learning to predict the personality traits of extension nodes. After getting all nodes in users’ personality data, it analyzes problems like whether there is a link between personality and user’s relationship or what is the relationship. Finally, calculations are made from four dimensions to determine the possibility of missing links or possible future links. Four dimensions are calculated as follows: similarity of user attributes, including location, follow tag, and other attributes; Network Structure Connection Tightness refers to the topology-based similarity with measures based on nodes and paths; Asymmetric Interaction Intensity includes one-way and two-way attention since the relationship of Weibo users is a directed network; Matching Degree of Personality Link Preference calculates the similarity between unlinked nodes of all nodes and their link preferences based on the link preference induced in the previous step.

### Personality Traits Prediction

This paper aims to explore whether personality traits play a role in link prediction and what kind of role they play. That is, to discuss the influence of personality traits on link prediction. To verify this idea, it needs to obtain the personality data of users in social networks first. As not all users can fill in the personality questionnaire, it is necessary to make personality prediction for users who cannot obtain the personality data. After obtaining the personality data, it analyzes the connection between personality and user followership, and then considers whether personality factors should be taken into account in the link prediction.

This paper chooses Weibo, one of the most popular social media in China, as the research object, and draws on the methods of personality prediction in domestic and foreign literature to comprehensively analyze users’ characteristics in social network, such as the characteristics of static attributes, text language, and dynamic behavior. Then, the authors use three commonly used machine learning algorithms to train personality prediction models, select the optimal model to predict the personality of the extension node, and analyze the personality data.

Among them, static attribute characteristics refer to the user’s long-term inherent attributes, usually not easy to change. The static attribute characteristics selected in this paper include characteristics like demographic characteristics (gender, registration time, age, and location), active display characteristics (a nickname and a personal profile), follow tag characteristics (the number of follow tags), and text language characteristics. This paper employs the tool of LIWC (linguistic inquiry and word count) to make a statistical analysis of the part of speech of blog posts and explores the relationship between users’ writing habits and personalities. In this paper, the Chinese LIWC dictionary is used as the standard for part-of-speech analysis and word frequency statistics, and word frequency is then stored in the feature set as part of a speech feature.

The dynamic behavior characteristics refer to the attributes that require indirect statistics or calculations and will change over time or over users’ performance. The dynamic behavior characteristics of this paper include counting characteristics (the total number of Weibo blog, Weibo level, number of fans, number of followers, number of photos, number of likes), credit rating (Sunshine Credit made by Weibo), active degree, and user influence.

The active degree indicates users’ different activeness when using Weibo. This paper mainly measures the user’s active degree from the perspective of posting frequency and like frequency. To simplify the calculation, it counts the frequency of blog posts along with the frequency of likes. Equation (1) shows how to calculate a user’s active degree.


(1) $${\text{Act}}_{i}=\frac{T\,o\,t\,a\,l_{i}^{w}+T\,o\,t\,a\,l_{i}^{l}}{D\,a\,y\,s_{i}}$$

$T\,o\,t\,a\,l_{i}^{w}$ is the total number of blogs posted by the user *i*, $T\,o\,t\,a\,l_{i}^{w}$ is the total number of likes of the user *i*, and *Days*_*i*_ is the total number of days that the user *i* uses the Weibo. The time specified in the formula is the number of days from the registration time to December 1, 2018, which is simplified according to the standard of 12 months a year and 30 days a month.

User Influence in social networks refers to the ability of a user’s opinions or behaviors to influence other users after being accepted by other users. The greater the influence is, the higher the degree of being followed, and it is the more likely for them to become leaders of a key opinion in social networks. The leadership temperament is also a manifestation of personality. This paper measures the influence of users through interactions among Weibo users. More retweets, comments, and likes indicate greater user influence. The following formula represents the influence of user *i*:


(2) $$\text{Inf}\,\left(i\right)=\frac{\sum_{w=1}^{{\mathrm{Toal}}_{i}^{k}}\left({\text{Num}}_{\mathrm{iw}}^{\mathrm{rep}}+{\text{Num}}_{\mathrm{iw}}^{\mathrm{com}}+{\text{Num}}_{\mathrm{iw}}^{\mathrm{lik}}\right)}{{\text{Total}}_{i}^{w}},$$

$N\,u\,m_{i\,w}^{r\,e\,p}$ refers to the number of user *i*’s *w*th blog, which has been reposted. $N\,u\,m_{i\,w}^{c\,o\,m}$ is the total number of comments of the *w*th blog posted by user *i*, including user *i*’s own reply. $N\,u\,m_{i\,w}^{l\,i\,k}$ is the number of likes of the *w*th blog posted by user *i*. $T\,o\,t\,a\,l_{i}^{w}$ is the total number of user *i*’s blogs.

In summary, a total of 113 features are collected and sorted out in this paper as the feature set of Weibo users, while only 149 seed nodes can be used as the training set. For cases with high feature dimensions but small sample data, it is necessary to reduce the dimension of the feature set while maintaining the validity of original data. This paper adopts the method of feature selection to reduce the dimension. The commonly used methods of feature selection include filtering and embedding. Filtering is a method easy to understand, which can control the number of features, but there are often correlations among features that can be redundant. Applying the method of embedding to the feature selection can solve the problem that filtering cannot do, but the number of features retained by this method is difficult to control, and the importance of retained features cannot be measured. Using filtering to express the correlation between features and target values can make up for the problem that the importance of candidate features cannot be measured. It can be seen that filtering and embedding have their own advantages and disadvantages, and they can complement each other. Therefore, this paper combines the correlation-based filtering method with the Lasso-based embedding method for feature selection. It applies Pearson correlation coefficient to filter irrelevant features, ensuring the importance of features, and then it uses Lasso to remove redundant features, namely the hybrid feature selection method.

### Four-Dimensional Link Prediction Model

#### Calculation of User Attribute Similarity

The user attribute information is the user’s personal background information, most of which is filled in by the user himself, including the user’s social background and hobbies. The attribute information of users in social networks is the basic information to identify users of nodes. Link prediction based on the similarity of user attributes is mainly based on the belief that users with similar attributes are more likely to establish social relationships. For example, users are more likely to establish relationships with people of similar geographical location and age (Xu, 2018, 2019).

This paper firstly measures the similarity of attributes between two users. The used user attributes are gender, age, location, sunshine credit, and follow tag, which is defined as *UP* = {*gen*,*age*,*loc*,*cre*,*fol*}. The specific value takes user *u* and user *v* as the examples. If user *u* and user *v* have the same gender, then it takes the equation of {*gen*(*u*) = 1,*gen*(*v*) = 1}; if the gender is different, then it takes the equation of {*gen*(*u*) = 1,*gen*(*v*) = 0}; if there is a missing value, then it takes the equation of {*gen*(*u*) = 0,*gen*(*v*) = 1}. When the age difference between user *u* and user *v* is less than or equal to 5 years old, then it takes the equation of {*age*(*u*) = 1,*age*(*v*) = 1}. When the age difference is over 5 years old, then it takes the equation of {*age*(*u*) = 1,*age*(*v*) = 0}. If there is a missing value, then it takes the equation of {*age*(*u*) = 0,*age*(*v*) = 1}. When the location of user *u* and user *v* is the same, then it takes the equation of {*loc*(*u*) = 1,*loc*(*v*) = 1}; otherwise, it takes the equation of {*loc*(*u*) = 1,*loc*(*v*) = 0}. If there are missing values, then it takes the equation of {*loc*(*u*) = 0,*loc*(*v*) = 1}. When user *u* and user *v* have the same credit rating, then it takes the equation of {*cre*(*u*) = 1,*cre*(*v*) = 1} but does not take the equation of {*cre*(*u*) = 1,*cre*(*v*) = 1} at the same time. If there is a missing value, it takes the equation of {*cre*(*u*) = 0,*cre*(*v*) = 1}. Comparing the follow tags of user *u* and user *v*, if there are some of the same follow tags, then it takes the number of the same tags of *fol*(*u*) and *fol*(*v*); otherwise, it takes the equation of {*fol*(*u*) = 1,*fol*(*v*) = 0}, and if there are missing values, then it takes the equation of {*fol*(*u*) = 0,*fol*(*v*) = 1}. This paper uses the Jaccard similarity coefficient to calculate the attribute similarity between users. Equation (3) shows how to calculate the similarity.


(3) $${\text{sim}}_{\mathrm{UP}}\,\left(u,v\right)=\frac{\left|\text{UB}\,\left(u\right)\,\cap \text{UB}\,\left(v\right)\right|}{\left|\text{UB}\,\left(u\right)\,\cup \text{UB}\,\left(v\right)\right|}=\frac{\text{p}}{\text{p}+\text{q}+\text{r}},$$

Equation (3) indicates the attribute similarity of two users by the proportion of the intersection between the attribute sets of user *u* and user *v* in their union. *p* represents the sum of values of the dimensions where user *u* and user *v* are not getting zero at the same time. *q* is the number of dimensions with user *u* values of 1 and user *v* values of 0. *r* is the number of dimensions with user *u* values of 0 and user *v* values of 1.

#### Calculation of Network Structure Connection Tightness

Firstly, the connection tightness is calculated based on the common neighbor node. For example, if A has a link with B and C, then A is called the common neighbor node of B and C. When two users have a common neighbor node, it means that they have a similar tendency to associate. For example, A and B follow C at the same time, and A follows D. Then, it is believed that B is likely to follow D. The more common neighbor nodes they have, the more similar their tendencies are. It turns out that the common neighbor node is the basic network structure element to measure the connection tightness between two nodes in the network.

In previous studies, there are many link prediction methods based on common neighbor nodes. For example, Common Neighbors (CN) directly uses the number of common neighbor nodes as a metric. Jaccard coefficient (JC) uses the ratio of common neighbor nodes to all neighbor nodes as the measuring index, and resource allocation (RA) and adamic-adar coefficient (AA) consider not only the number of common neighbors but also the degree of each common neighbor node. The latter method has better prediction effect, but it often offsets the importance of common neighbor nodes. For example, A and B have multiple common neighbor nodes with high degree and high correlation, which means that the degrees of these neighbor nodes are mainly contributed to other neighbor nodes instead of A and B. At this time, the connection tightness calculated by applying RA and AA is often lower than the actual value. Therefore, this paper uses an improved index to calculate the connection closeness between nodes based on common neighbors, and equation (4) shows how to calculate it.


(4) $$\text{con}\,\left(u,v\right)=\sum_{y\in \Gamma \,\left(u\right)\,\cap \Gamma \,\left(v\right)}\frac{1}{\left|\Gamma \,\left(y\right)\right|}\;\times \left(\frac{\left|\Gamma \,\left(u\right)\,\cap \Gamma \,\left(y\right)\right|+\left|\Gamma \,\left(v\right)\,\cap \Gamma \,\left(y\right)\right|}{2}\right),$$

In equation (4), Γ(*u*) represents the set of neighboring nodes of user *u*, |Γ(*u*)| represents the number of all neighbor nodes of user *u*, Γ(*u*)∩Γ(*v*) represents the common neighbor nodes between user *u* and user *v*, and |Γ(*u*)∩Γ(*v*)| represents the number of common neighbor nodes. In addition, in order to avoid the situation of $\frac{\left|\Gamma \,\left(u\right)\,\cap \Gamma \,\left(y\right)\right|+\left|\Gamma \,\left(v\right)\,\cap \Gamma \,\left(y\right)\right|}{2}$ being less than 1, it will be replaced with the value of 1 when values are less than 1.

Then, the connection tightness is calculated based on the path. In the network structure, in addition to common neighbor nodes, link edges between nodes are also important factors to describe the network structure. The existing edge-based measure methods include local path (LP) measurement based on local paths and Katz measurement based on global paths. In this paper, since the path of more than three hops in social networks is difficult for users to detect, there will be no impact on the link relationship between users. Therefore, the LP-based measurement is used to measure the tightness of the path-based connection:


(5) $$\text{LP}\,\left(u,v\right)=\frac{{\text{A}}_{u,v}^{2}+\alpha \,{\text{A}}_{u,v}^{3}}{{\text{A}}_{u,v}},$$

In equation (5), $A_{u,v}^{2}$ represents an adjacency matrix with a length of 2 between user *u* and user *v*, and $A_{u,v}^{3}$ is an adjacency matrix with a length of 3, which is also equal to the number of paths with a length of 2 and 3 between user *u* and user *v*, respectively. A path of length 2 is more influential than a path of length 3. Therefore, α is added to the formula as the attenuation coefficient. In the calculation of this paper, α = 0.3 is used, and *A*_*u,v*_ is the number of all paths between user *u* and user *v*. In addition, it is worth noting that it chooses Sina Weibo as the social network in this paper. Its characteristics are that the edges in the network are directed edges or bidirectional edges, so special attention should be paid to the direction of the edges when calculating the number of edges between two points. It is stipulated that it can be counted as a path only when all edges have consistent directions. Therefore, the tightness of the network structure connection between users *u* and *v* in this paper is shown in Equation (6).


(6) $$\text{com}\,\left(u,v\right)=\frac{\text{con}\,\left(u,v\right)+\text{LP}\,\left(u,v\right)}{2},$$

#### Calculation of Asymmetric Interaction Intensity

In social networks, in addition to speculating the possibility of non-existing links based on the attributes between users and existing links, the interaction between users should also be put into consideration. They have already interacted with each other, so the possibility of a link between interactive users is obviously greater than that without interaction. At the same time, as in social networks like Weibo, attention and interaction are directional. When the interaction is unilateral, a one-way follow in the same direction may be generated. When both parties have strong interactions, a two-way follow may be generated. So, when calculating the impact of interaction on user relationships, we should not only consider the existence of interactions or the number of interactions but also the direction of interactions. The initiators and recipients of directed interactions have different perceptions of their interaction intensity, so the interaction intensity in directional social networks is called an asymmetric interaction intensity. Use *act*_*u*→*v*_(*b*_*i*_) to denote the interaction intensity of user *u*, initiating interactive action *b*_*i*_ on user *v*. The calculation method is shown in equation (7).


(7) $${\text{act}}_{u\to v}\,\left({\text{b}}_{i}\right)=\frac{{\text{n}}_{u\to v}^{b_{i}}}{{\text{n}}_{u\to }^{b_{i}}+1},$$

In equation (7), $n_{u\to v}^{b_{i}}$ indicates the number of time that user *u* initiated the interactive behavior *b*_*i*_ for user *v*, $n_{u\to \cdot }^{b_{i}}$ indicates the number of time that user u initiated the interactive behavior *b*_*i*_ for all users, and the denominator plus 1 is to prevent the situation where the denominator is 0. The interaction behaviors considered in this paper are retweets, comments, and likes, so the range of *b*_*i*_ is *b*_*i*_ ∈ {*rep*,*com*,*lik*}. As the influence of the interaction behavior is *rep* > *com* > *lik*, the interaction intensity between user *u* to user v is calculated as shown in equation (8).


(8) $${\text{act}}_{u\to v}={\text{act}}_{u\to v}\,\left(\mathrm{rep}\right)+\frac{2}{3}\,{\text{act}}_{u\to v}\,\left(\mathrm{rev}\right)+\frac{1}{3}\,{\text{act}}_{u\to v}\,\left(\mathrm{lik}\right),$$

When considering the probability that user *u* points to user *v*, it needs to calculate the interaction intensity of user *u* to user *v*, which refers to *act*_*u→v*_. Similarly, when considering the probability that user *v* points to user *u*, the interaction intensity between user *u* and user *v*can be *act*_*v→ u*_.

#### Calculation of Matching Degree of Personality Link Preference

After personality prediction, this paper explores the association between personality and social relationships, proves the existence of personality link preferences, and calculates the link preferences of specific personality (as shown in Table 7). Therefore, these conclusions are directly adopted in this section. When judging whether user *u* will pay attention to user *v* or not, it firstly determines the category to which each dimension of user *u* belongs and its corresponding link preference personality, and then it calculates the link preference vector of user *u*, as shown in equation (9).


(9) $${\text{pre}}_{u}=\frac{\begin{array}{c} {\text{O}}_{u}\cdot {\text{pre}}_{O}\,\left(u\right)+{\text{C}}_{u}\cdot {\text{pre}}_{C}\,\left(u\right)+{\text{E}}_{u}\cdot {\text{pre}}_{E}\,\left(u\right)+{\text{A}}_{u}\\ \cdot {\text{pre}}_{A}\,\left(u\right)+{\text{N}}_{u}\cdot {\text{pre}}_{N}\,\left(u\right) \end{array}}{{\text{P}}_{u}},$$

In equation (9), *O*_*u*_ is the score of user *u* on openness, *C*_*u*_ is the score of user *u* in terms of conscientiousness, *E*_*u*_ is the score of user *u* on extraversion, *A*_*u*_ is the score of user *u* on agreeableness, *N*_*u*_ is the score of user *u* on neuroticism, *pre*_*O*_(*u*) is the link preference of the category of user *u* openness, *pre*_*C*_(*u*) is the link preference of the category of user *u* conscientiousness, *pre*_*E*_(*u*) is the link preference of the category of user *u* extraversion, *pre*_*A*_(*u*) is the link preference of the category of user *u* agreeableness, *pre*_*N*_(*u*) is the link preference of the category of user *u* neuroticism, *pre*_*O*_(*u*), *pre*_*C*_(*u*),*pre*_*E*_(*u*), *pre*_*A*_(*u*), *pre*_*N*_(*u*), showing the link preference values in n can be obtained by referring to Table 7, and *P*_*u*_ is the sum of the user *u*’s scores in the five dimensions of personality. It calculates the matching degree $m\,a\,t_{v}^{u}$ of link preferences of user *v* and user *u* according to the calculation method shown in equation (10).


(10) $${\text{mat}}_{v}^{u}=\frac{{\text{pre}}_{u}^{O}}{{\text{pr}}_{u}}\cdot \frac{1}{\left|{\text{O}}_{v}-{\text{pre}}_{u}^{O}\right|}+\frac{{\text{pre}}_{u}^{C}}{{\text{pr}}_{u}}\cdot \frac{1}{\left|{\text{C}}_{v}-{\text{pre}}_{u}^{C}\right|}+\frac{{\text{pre}}_{u}^{E}}{{\text{pr}}_{u}}\cdot \frac{1}{\left|{\text{E}}_{v}-{\text{pre}}_{u}^{E}\right|}+\frac{{\text{pre}}_{u}^{A}}{{\text{pr}}_{u}}\cdot \frac{1}{\left|{\text{A}}_{v}-{\text{pre}}_{u}^{A}\right|}+\frac{{\text{pre}}_{u}^{N}}{{\text{pr}}_{u}}\cdot \frac{1}{\left|{\text{N}}_{v}-{\text{pre}}_{u}^{N}\right|}$$

In equation (10), $p\,r\,e_{u}^{O}$ represents the openness value in the link preference vector of user *u*, similarly, $p\,r\,e_{u}^{C}$ is the conscientiousness value in the link preference vector of user *u*, and $p\,r\,e_{u}^{E}$ is the extraversion value in the link preference vector of user *u* values, $p\,r\,e_{u}^{A}$ is the value of agreeableness in the user’s link preference vector, $p\,r\,e_{u}^{N}$ is the value of neuroticism in the link preference vector of user *u*, *pr*_*u*_ is the sum of the personality trait scores in the link preference vector of user *u*, *O*_*v*_ is the score of user *v* on openness, *C*_*v*_ is the score of user *v* on conscientiousness, *E*_*v*_ is the score of user v on extraversion, *A*_*v*_ is the score of user *v* on agreeableness, and *N*_*v*_ is the score of user *v*on neuroticism. The score on $m\,a\,t_{v}^{u}$ being higher indicates that the higher the matching degree between the personality of user *v* and the link preference personality of user *u*, the more likely it is to produce a link from user *v* to user *u*.

#### Construct Four-Dimensional Link Prediction Models for Social Networks

To sum up, this paper fully considers the influence of such four dimensions as user attribute similarity, network structure connection tightness, asymmetric interaction intensity, and the matching degree of personality link preference, and this paper integrates these four dimensions to form a new comprehensive link prediction model of social network. The link prediction calculation model calculate the link probability *link*_*u→v*_ of each edge in the set of unconnected edges and ranks the edges in descending order according to their scores. The higher the sorting edge is, the more likely it is to generate links, that is, the greater *link*_*u→v*_ is, the more likely it is to produce link *u*→*v*. The calculation method of link prediction probability *link*_*u→v*_ is shown in equation (11).


(11) $${\text{link}}_{u\to v}=\alpha \cdot {\text{sim}}_{\mathrm{UP}}\,\left(u,v\right)+\beta \cdot \text{com}\,\left(u,v\right)+\gamma \cdot {\text{act}}_{u\to v}+\delta \cdot {\text{mat}}_{v}^{u},$$

In equation (11), α, β, γ, δ are the weight values of each of the four dimensions, respectively, and they satisfy such five conditions as 0 < α < 1, 0 < β < 1, 0 < γ < 1, 0 < δ < 1, and α + β + γ + δ = 1. The idea of determining the weight value in this paper is to take 0.1 as the unit length of the weight, substitute all the collocation values of the four weights into the model for calculation, and then select the optimal corresponding weight according to the evaluation index of the model so as to determine the final expression of FDLPM proposed in this paper. The calculation process of the four-dimensional integrated link prediction model of social network is shown in Algorithm 1.

**Algorithm 1:** Calculation steps of the FDLPM model.

**INPUT:** User attribute data set UP, follow relation matrix UG, interaction behavior list UA, user personality data UT
**OUTPUT:** Sequence L of Linked Probability of Unconnected Edges
**Step 1:** Process the user attributes according to the rules described in Section “Calculation of User Attribute Similarity,” and the attribute matrices corresponding to *gen*, *age*, *loc*, *cre*, and *fol* are respectively, constructed;
**Step 2:** Convert the UG into *G*(*V*,*E*) and divide the edge set *E* equally into 10 parts, of which 9 parts are used as the training set *E^T^*, and the other one as the test set *E^P^*;
**Step 3:** Hide the *E^P^*− related relationship from the *G*(*V*,*E*), that is *G*′(*V*,*E*−*E^P^*), traverse the *G*′(*V*,*E*−*E^P^*), find and record the number of common neighbor nodes between nodes and the number of paths with lengths of 2 and 3;
**Step 4:** Calculate the metric values of all sides in *U*−*E^T^* in four dimensions according to formulas (3), (6), (8), (10);
**Step 5:** According to the weight traversal method described of Algorithm 2, calculate the link probability of all edges in *U*−*E^T^* by combining the four dimensions with different weights;
**Step 6:** Compare the test set *E^P^* to calculate the AUC and precision of the parameter prediction results of each group, and record the optimal parameter group;
**Step 7:** Replace a group of a training set and a test set, and skip back to Step 3 until all edges have done test sets, average the ten groups of optimal weights, and substitute the average optimal weight into the FDLPM model;
**Step 8:** Traverse the *G*(*V*,*E*) to find and record the number of common neighbor nodes between nodes and the number of paths with lengths of 2 and 3;
**Step 9:** Calculate the metric values for all four sides of *U-E* in accordance with formulas (3), (6), (8), (10);
**Step 10:** Calculate the link probability of the edges in *U-E* with the model obtained in step 7, and sort it in descending order according to the probability to obtain *L*.

Algorithm 2 lists the schematic code of the weight training process in FDLPM. The purpose of weight training is to train the optimal weight combination for different experimental data sets to improve the prediction accuracy of the model.

**Algorithm 2:** The weight training algorithm of the FMLPM model.

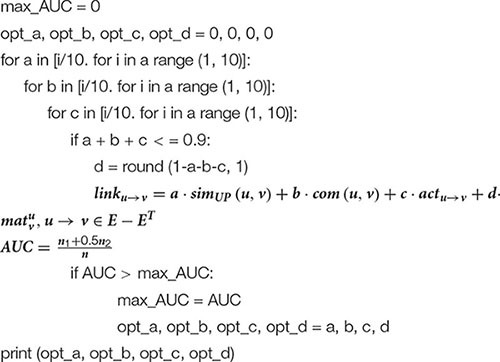

### Experimental Analysis

#### Data Set

In this paper, Sina Weibo is selected as the data source for the experiment. There are 217 users who filled in the personality questionnaire. After crawling the Weibo data, it is necessary to screen the blog content. Users who have the following situations will be filtered out: users whose number of blog is less than 30, users who have registered for less than 6 months, users with more than half of similar blog posts (e.g., “repost,” ads, lottery draw, punch the clock, etc.). Finally, 149 valid Weibo users’ IDs are screened out in this experiment, and 149 corresponding users are selected as the seed nodes for this experiment with 52 males (34.9%) and 97 females (65.1%), mainly aging from 19 to 26. The total number of blogs is 114,384, and it is 767.6 blogs *per capita*. Then, crawl the follow list and follower list of the seed nodes (while crawling the follow list, the follow tags are obtained and stored in the user’s basic information table), and randomly select five users from the follow list and follower list as extension nodes. For that step, it preliminarily selects 1,490 extension nodes; remove the extension nodes that duplicate the seed nodes, and filter the extension nodes according to the criteria of filtering seed nodes; finally, retain 1,196 extension nodes. Then, the basic information and blog contents of extension nodes are further crawled, and these data, along with the data of seed nodes, constitute the data set of personality prediction. Finally, the experimental data set contains a total number of 1,345 Sina Weibo users with 594 male users (44.16%) and 751 female users (55.84%), mainly aging from 18 to 48 years old. The total number of blogs is 1,745,224, and 1,297.6 blogs *per capita*. By comparing the same attributes of seed nodes, it is found that seed nodes are mostly similar and are distributed in a concentrated way as users of seed nodes are all spread by the author himself and his friends. However, after adding the extension nodes, the distribution of users in the whole data set is more universal. In order to verify the accuracy of the link prediction method proposed in this paper and estimate the relevant parameters in the algorithm, this research uses RapidMiner software to randomly divide the collected data into a training set and a test set, and randomly divide the user data into a 9:1 ratio. For the training set and the test set, k-fold cross-validation is performed to ensure the reliability of experimental results of this study.

### Experimental Results

#### Personality Prediction Experiment

In the personality prediction experiment, such commonly used regression models as linear regression, random forest, and decision tree algorithms are selected to do model training, respectively, and the method in python integration library sklearn (Bao et al., 2020) is used to realize the model training. The experimental environment configuration of this study is shown in Table 1.

**TABLE 1:** Experimental environment configuration.

Operating					
System	Processor	CPU	Core	RAM	Software
Win 10	Intel Core i5-8265U	3.4GHz	8 cores	8G	PyCharm 2017

**FIGURE 3:**
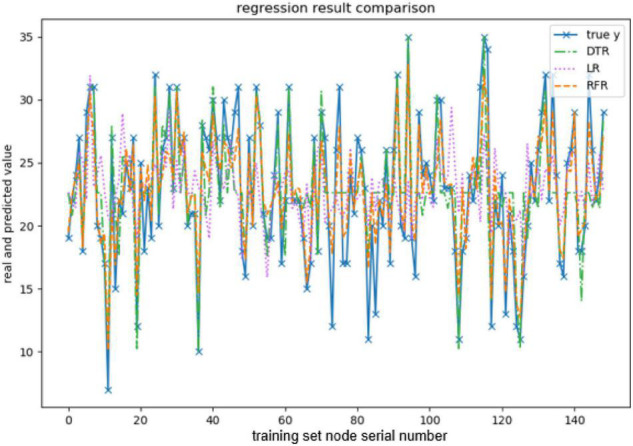
Comparison of openness predicted values.

**FIGURE 4:**
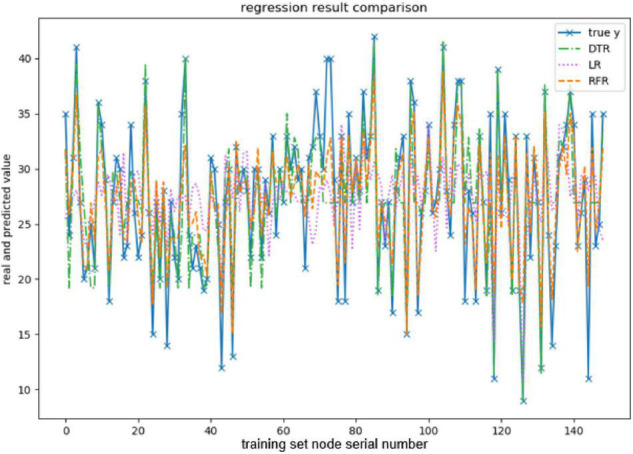
Comparison of the predicted values of conscientiousness.

**FIGURE 5:**
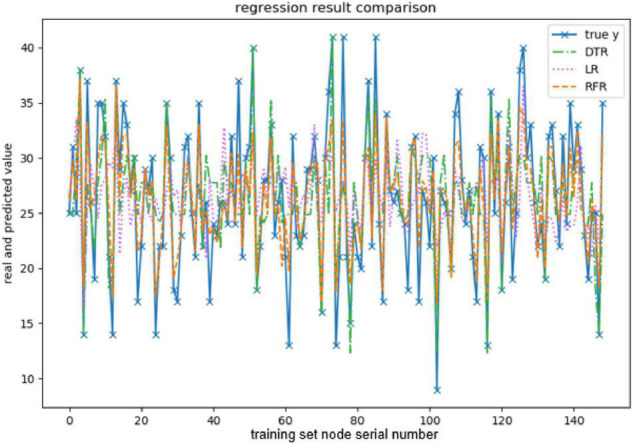
Comparison of extraversion-predicted values.

**FIGURE 6:**
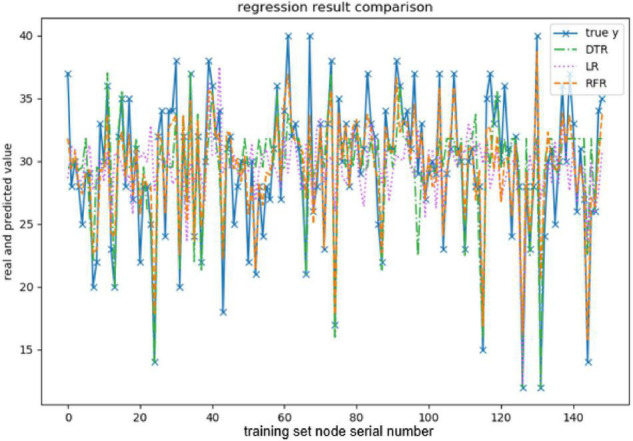
Comparison of agreeableness-predicted value.

**FIGURE 7:**
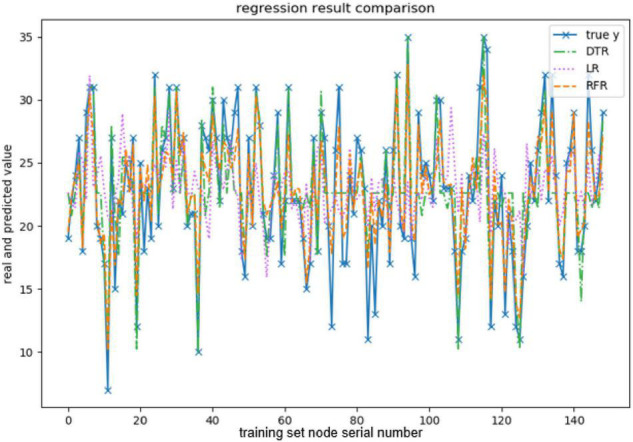
Comparison of neuroticism-predicted values.

When evaluating the effect of the training model, this paper takes explained variance (EV), mean absolute error (MAE), Mean squared error (MSE), and R-squared (*R*^2^) as evaluation indexes, and adopts 10-fold cross-validation to be the verification method. The effect of the model training is shown below, and the results are shown in Figures 3–7 and Tables 2–6.

**TABLE 2:** Regression index of the openness model.

	EV	MAE	MSE	*R* ^2^
DTR	0.639955	3.661632	23.710683	0.639955
LR	0.319572	5.134217	40.223183	0.319572
RFR	0.821502	2.278523	9.212013	0.821265

**TABLE 3:** Regression index of the conscientiousness model.

	EV	MAE	MSE	*R* ^2^
DTR	0.753592	2.847626	17.335559	0.753592
LR	0.2303896	5.012247	39.839996	0.303896
RFR	0.815564	2.338926	9.271208	0.814738

**TABLE 4:** Regression index of the extroversion model.

	EV	MAE	MSE	*R* ^2^
DTR	0.595300	3.702011	23.164957	0.595300
LR	0.318499	4.890109	36.787673	0.318499
RFR	0.804836	2.251678	8.975906	0.804440

**TABLE 5:** Regression index of the agreeableness model.

	EV	MAE	MSE	*R* ^2^
DTR	0.590645	2.754499	13.119245	0.590645
LR	0.322494	3.918414	25.879397	0.322494
RFR	0.839393	1.716778	5.147248	0.839392

**TABLE 6:** Regression index of the neuroticism model.

	EV	MAE	MSE	*R* ^2^
DTR	0.676955	2.720418	13.816487	0.676955
LR	0.320247	4.098513	26.446232	0.320247
RFR	0.840250	1.814094	5.221275	0.840131

**TABLE 7:** A link preference of personality traits.

	Openness	Conscientiousness	Extraversion	Agreeableness	Neuroticism
High openness (*O*_*high*_)	39.81	24.84	27.69	27.07	18.15
Low openness (*O*_*low*_)	38.16	31.19	22.41	29.65	23.65
High conscientiousness (*C*_*high*_)	39.42	31.01	26.54	28.64	21.01
Low conscientiousness (*C*_*low*_)	37.60	26.37	27.28	30.45	22.57
High extraversion (*E*_*high*_)	37.35	27.88	26.19	29.83	22.25
Low extraversion (*E*_*low*_)	39.58	30.44	23.92	28.86	20.69
High agreeableness (*A*_*high*_)	39.7	28.03	26.74	31.01	23.01
Low agreeableness (*A*_*low*_)	38.32	29.09	26.26	28.02	20.99
High neuroticism (*N*_*high*_)	39.41	29.69	30.49	30.71	21.85
Low neuroticism (*N*_*low*_)	39.27	27.41	24.91	28.37	22.81

The above are the trainings of five personality prediction models. There is an intuitive comparative diagram of the predicted value and the true value, and an evaluation index of the regression model. EV is equal to 1 minus the ratio of the variance of the error between the predicted value and the real value to the variance of the real value. The closer the value is to 1, the better it is. MSE is the ratio of the square sum of the deviation between the observed value and the true value and the number of observations, which is the most commonly used loss function in linear regression. In the regression process, it is supposed to maintain the value of the loss function as small as possible. The smaller the value of MSE is, the more accurate the prediction model will be in describing the experimental data. MAE is the average of the absolute error between the predicted value and the true value. R^2^ is the ratio of the model’s predicted errors to the average value of the observation. The value of R^2^ is between 0 and 1. The closer it is to 1, the better the regression fitting effect is. According to the above regression indexes, it can be seen that, in the five personality features, the random forest regression model performs the best, and the linear regression is the worst, followed by the decision tree, because the random forest has the highest degree of fit and the smallest prediction error. Therefore, we chose the random forest model combined with the previously selected feature set as the model for personality prediction in this paper.

**FIGURE 8:**
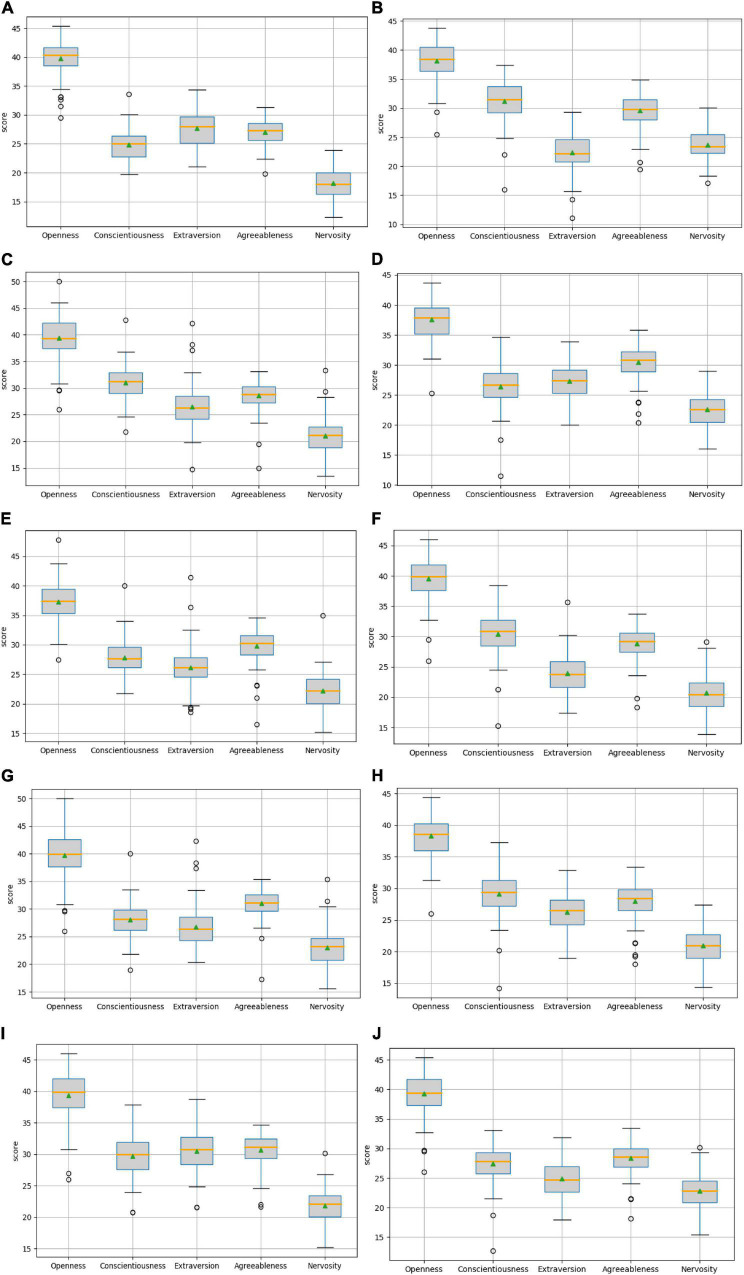
The personality distribution of follow users for each type is shown below: **(A)** The personality distribution of follow of users with high openness; **(B)** The personality distribution of follow of users with low openness; **(C)** The personality distribution of follow of users with high conscientiousness; **(D)** The personality distribution of follow of users with low conscientiousness; **(E)** The personality distribution of follow of users with high extraversion; **(F)** The personality distribution of follow of users with low extraversion; **(G)** The personality distribution of follow of users with high agreeableness; **(H)** The personality distribution of follow of users with low agreeableness; **(I)** The personality distribution of follow of users with high neuroticism; **(J)** The personality distribution of follow of users with low neuroticism.

Users who score high on the same personality trait will show different personalities and behaviors than users who score low. Therefore, when discussing the influence of personality traits on their behaviors, it is supposed to divide users into two groups: one with high trait scores and the other with low trait scores. This paper uses the arithmetic average of each personality trait as the basis of division. Users with a score greater than the average are classified as high, and users with a score lower than the average are classified as low. In the data set of this paper, there are 703 users with high openness, 642 users with low openness, 728 users with high conscientiousness, 617 users with low conscientiousness, 650 users with high extraversion, 695 users with low extraversion, 722 users with high agreeableness, 623 users with low agreeableness, 743 users with high neuroticism, and 602 users with low neuroticism. The set composed of the above users is called {*O*_*high*_,*O*_*low*_,*C*_*high*_,*C*_*low*_,*E*_*high*_,*E*_*low*_,*A*_*high*_,*A*_*low*_,*N*_*high*_,*N*_*low*_}. In order to investigate whether personality traits affect the following relationship between users, that is, whether different personality traits correspond to different link preferences, we observed the personality traits of their follow nodes in turn according to the division of users above. We matched the following all the user IDs in the above set, organize their personality trait scores into files, and then draw a box plot to reflect their distribution, which has been shown below (Figure 8).

Figure 8 shows the personality distributions of all follow users with different personality traits users and summarize the regularity. The upper and lower edges of the gray rectangle are the upper quartile and the lower quartile of the corresponding personality trait data. The orange horizontal line in the middle is the median, the small green triangle indicates the position of the average value, and the small circle indicates the position of the outlier. The biggest advantage of the box plot is that it is not affected by the outlier, and the discrete distribution of data can be described in a relatively stable way. Comparing the above figure, it can be seen that the two groups of people with high and low personality traits have different personality distributions of their followers. For example, users with high openness are less conscientious than those with low openness, while their extroversion is relatively high. At the same time, there are differences in the attention of different personality trait dimensions, for example, the attention of high openness is more unified than that of high conscientiousness. In this paper, these differences are summarized as link preferences corresponding to personality traits, and these preferences are shown by the average value of personality traits of people followed. According to these rules, the link preferences of users with various personality traits are shown in Table 7.

#### A Comparison Experiment of Link Prediction

First of all, since the prediction model is always difficult to avoid prediction errors, we generally chose to verify the accuracy in the existing links to measure the accuracy of the model. The set E of known edges is randomly divided into two parts with the training set *E^T^* and the test set *E^P^*, and then it gets *E^T^*∪*E^P^* = *E* and *E^T^*∩*E^P^* = ∅. The advantage of the random sampling validation is that the proportion of training segmentation does not depend on the number of iterations. However, with this approach, some links may not appear in the validation set, while others may be selected multiple times, which may lead to some deviations. This shortcoming can be overcome by using multiple cross-validations. It randomly divides the observed links into K subsets and selects one subset each time as the test set. The remaining k-1 subsets constitute the training set. Then, it repeats the cross-validation process for K times with each subset used as the verification set. By doing so, all links are used for training and validation, and each link is predicted one time. Obviously, the larger the K is, the smaller the statistical deviation and the greater the calculation amount will be. This paper adopts 10-fold cross verification.

The commonly used evaluation indexes for link prediction are area under the receiver operating characteristic curve (AUC) and precision. AUC is proposed based on the receiver operating characteristic curve (ROC), which represents the area above the coordinate axis and below the ROC curve. AUC can measure the accuracy of the prediction model as a whole, which refers to the probability that the link probability of the edge randomly selected from the test set is greater than that of the edge selected from the non-existent edge set. The specific calculation process is as follows: randomly choose an edge from the test set *E^P^*and the non-existent set *U-E* as *e* and *e*′, respectively, and compare the link probability of two edges with *link*_*e*_ and *link*_*e’*_. If *link*_*e*_ > *link*_*e*′_, then add up to 1. If *link*_*e*_ = *link*_*e*′_, then add up to 0.5. If *link*_*e*_ < *link*_*e*′_, then add up to 0. It needs to make such random choices for *n* times. The number of cases satisfying *link*_*e*_ > *link*_*e*′_ is recorded as *n*_*1*_, the number of cases satisfying *link*_*e*_ = *link*_*e*′_ is recorded as *n*_*2*_, and the number of cases satisfying *link*_*e*_ < *link*_*e*′_ is recorded as *n*_*3*_. Formula (12) shows the calculation of AUC:


(12) $$\text{AUC}=\frac{{\text{n}}_{1}+0.5\,{\text{n}}_{2}}{\text{n}},$$

If all link probabilities are generated randomly, the AUC value is 0.5. Therefore, it is generally required that the AUC of the prediction model should be greater than 0.5. The larger the AUC is, the better the prediction effect of the model will be. In addition, in order to make the calculation result of AUC as accurate as possible, there are a total of 10,477 edges in the data set, so *n* = 1,047.

Different from AUC, the precision only considers the prediction accuracy of the first m edges of the link probability sequence L, which is defined as the proportion of the first m edges belonging to the test set after the link probability is sorted. Formula (13) shows how to calculate it.


(13) $$\text{Precision}=\frac{{\text{n}}_{L}}{\text{m}}$$

Among the first m edges of sequence L, n edges belong to *E^P^*. Obviously, the value of precision varies with the value of *m*, and when *m* is given, the higher the value of precision is, the better it will be, and usually, it takes *m* = 100.

**FIGURE 9:**
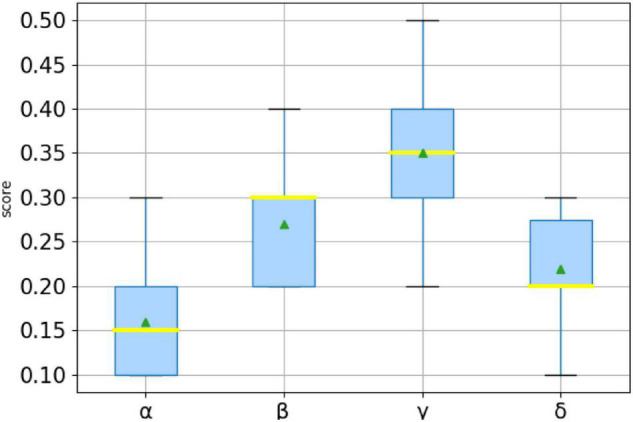
Distribution of optimal weights.

Next, the FDLPM model parameters will be trained according to Algorithm 2 described in Section “Construct a FDLPM for Social Networks.” Since the experiment in this paper adopts 10-fold cross-validation, the calculation process of Algorithm 2 will be performed 10 times. After combining the outputs of ten optimal weights, it takes the average value, and then takes the average optimal weight as the final weight value of the comprehensive link prediction model. The distribution of the ten groups of optimal weights outputted in the weight training process of the data set in this paper is shown in Figure 9.

Figure 9 is a box plot of ten groups of optimal weights, where the horizontal yellow line represents the median and the small green triangle marks the location of the average. The value of α between [0.1,0.3], the upper quartile was 0.1, the median was 0.15, the lower quartile was 0.2, and the average was 0.16; The value of βbetween [0.2,0.4], the upper quartile was 0.2, the median was 0.3, the lower quartile was 0.3, and the average was 0.27; The value of γ between [0.2,0.5], the upper quartile was 0.3, the median was 0.35, the lower quartile was 0.4, and the average was 0.35; The value of δ between [0.1,0.3], the upper quartile was 0.2, the median was 0.2, the lower quartile was 0.275, and the average was 0.22. Then the average optimal weight is α = 0.16, β = 0.27, γ = 0.35, δ = 0.22.

The values of the above optimal weights are all within a reasonable range. At the same time, the rationality of the FDLPM model proposed in this paper has been proved. And the value of weights has a tendency of γ > β > δ > α, which indicates that the interaction behavior in social networks is an important factor that can influence link prediction, and that the personality link preference also has a certain influence on link prediction and the user attribute similarity has the least influence. The reason for that situation might be that users choose not to fill in or fill in false information to protect personal data.

**FIGURE 10:**
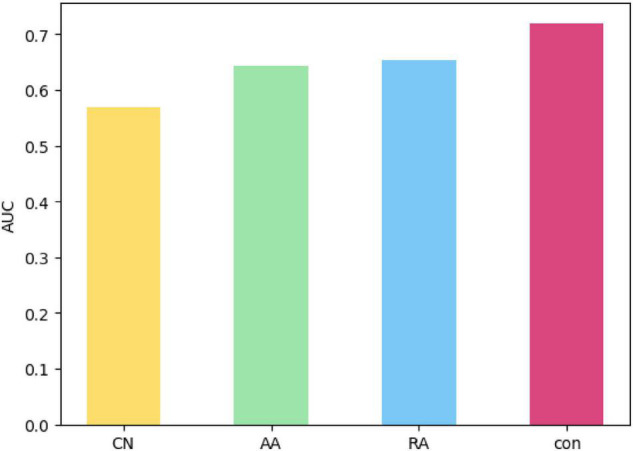
AUC comparison of different metrics.

In this paper, a comparative experiment is set up for the connection tightness of network structure. In order to verify the impact of the connection tightness of network structure based on common neighbor nodes applied in this paper on link prediction, it compares with the three metrics of CN, AA, and RA with the follow three equations: *CN*(*u*,*v*) = |Γ(*u*) + Γ(*v*)|,$A\,A\,\left(u,v\right)=\sum_{z\in \Gamma \,\left(u\right)\,\cap \Gamma \,\left(v\right)}\frac{1}{\text{log}\,\left|\Gamma \,\left(z\right)\right|}$ and $R\,A\,\left(u,v\right)=\sum_{z\in \Gamma \,\left(u\right)\,\cap \Gamma \,\left(v\right)}\frac{1}{\left|\Gamma \,\left(z\right)\right|}$. Comparative experiments use the same data set, and take AUC and precision as evaluation indexes. Figure 10 shows the average value after 10-fold cross-validation.

Figure 10 gives the AUC values of CN, AA, RA, as well as the *con* proposed in this paper based on the metrics of common neighbor nodes for link prediction in the same data set. The order of AUC value shown in the Figure 10 is that CN is less than AA, less than RA, and less than *con*, meaning that its prediction effect is sequentially improved.

**FIGURE 11:**
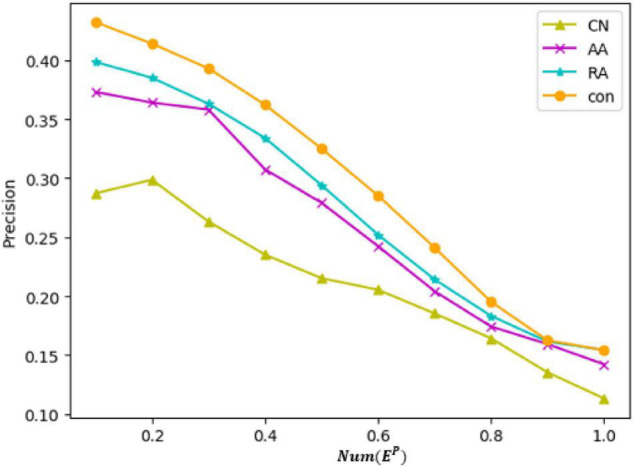
Precision comparison of different metrics.

Figure 11 shows the precision of the four metrics compared in the same figure, where the ordinate represents the value of precision and the abscissa represents the ratio of the sequence length m considered in the calculation of the precision to the *Num*(*E^P^*) (the total number of edges in the test set). Table 8 represents the precision of the prediction results of each metric when the value of m reaches 100. The chart shows that, as the value of m increases, the precision of each metric decreases, but the precision of *con* is always higher than that of other measurement methods. The results of the link prediction given by AUC and the precision prove that the prediction effect of *con* is better than other metrics. *Con* can be used as a metric to show the tightness of network connections between nodes. The reasons lie in that AA and RA consider not only the number of the common neighbor nodes but also the degree of common neighbor nodes and give relatively small weights to nodes with a greater degree compared with CN. The results prove that the prediction effect of AA and RA is better than that of CN. The *con* metric in this paper is an improvement of RA. When considering the degree of nodes, it is necessary to put into consideration the number of common neighbors between nodes and common neighbor nodes into consideration, which helps solve the problem of reducing the impact on the number of common neighbors due to the large degree and small weight. Thus, the result of *con* is better than AA and RA.

**TABLE 8:** When *m* = 100 is the precision value of each metric.

	CN	AA	RA	con
Precision	0.27	0.36	0.39	0.43

Finally, we set up experiments to verify the value of asymmetric interaction intensity and the matching degree of personality link preference in predicting social network links. The FDLPM model constructed in this paper not only considers the similarity of user attributes and the topological relationship between users, which refers to the tightness of the network connection often found in previous models, but also proposes the calculation of two dimensions of asymmetric interaction intensity and inter-user personality link preference matching for directed networks. To verify the role of these two dimensions in the whole link prediction model, we carried out a comparative experiment of three models. The two-dimensional link prediction model (APLP-2), only considering user attribute similarity and network structure connection tightness, uses the weight training method similar to Algorithm 2 to train model weights with AUC as the evaluation index, and the weights trained in this paper are 0.32 and 0.68, respectively. The three-dimensional link prediction model (APLP-3) takes into account three dimensions like user attribute similarity, network structure connection tightness, and asymmetric interaction intensity, and uses AUC as the evaluation index to train model weights. The weights trained in this paper are 0.24, 0.31, and 0.45, respectively. The four-dimensional link prediction model (APLP-4) considers such dimensions as the similarity of user attributes, network connection tightness, asymmetric interaction intensity, and the matching degree of personality link preference at the same time, with weights of 0.16, 0.27, 0.35, and 0.22, respectively. In this paper, the above model is applied to the same data set for experiments, and the listed result indicators are the average value of the 10-fold cross-validation. The results of the comparative experiments are as follows.

**FIGURE 12:**
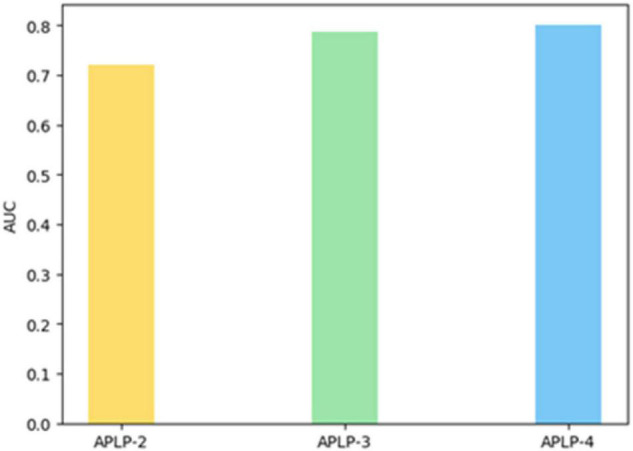
AUC comparison of different models.

Figure 12 shows the AUC values of the three link prediction models in the same data set. The AUC values shown in this figure show that ALPL-2 is less than ALPL-3 and less than ALPL-4, indicating that the prediction effect is improved in turn.

**FIGURE 13:**
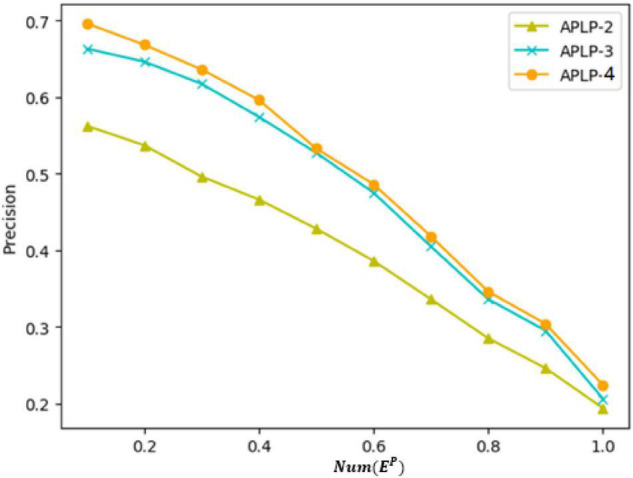
Precision comparison of different models.

Figure 13 shows the precision of three models for comparison in the same figure, where the ordinate represents the value of precision and the abscissa represents the ratio of the sequence length m considered in the calculation of precision to the *Num*(*E^P^*) (the total number of edges in the test set). Table 9 shows the precision of the prediction results of each model when the value of m reaches 100. It can be observed from the chart that the precision of each measurement shows a downward trend as the value of m increases. Based on the experimental results of AUC and the precision described above, the precision of the APLP-3 model is significantly improved, compared with the APLP-2 model, indicating that the asymmetric interaction intensity proposed in this paper has practical significance for link prediction in social networks. Compared with the three-dimensional link prediction model and the four-dimensional link prediction model, the precision of prediction is improved a little, indicating that the link preference matching degree based on personality preference is also effective. However, the precision is not greatly improved. This is because there is little difference in link preferences among different personalities, and most of the personality data in this paper are predicted, and there is an error in the personality data themselves, which affects the calculation of link preference matching. Also, when the APLP-3 and APLP-4 models in Figure 13 are smaller, the APLP-3 and APLP-4 models show a slower decline in accuracy than that of the APLP-2 models, which indicates that the APLP-3 or APLP-4 models proposed in this paper have better prediction effects when focusing on the link probability of the first *n* edges.

**TABLE 9:** When *m* = 100 is the precision value of each model.

	APLP-2	APLP-3	APLP-4
Precision	0.56	0.66	0.69

## Conclusion

The main research of this paper contains the following steps. Firstly, use Sina Weibo data to train a personality prediction model suitable for the Chinese context, optimize the design of the characteristic set, and combine filtering and embedding characteristic selection methods. Then, apply the trained personality prediction model to the extension node. After analyzing the personality data, it is found that there is a difference in the distribution of personality in social network users’ following relationships. This paper defines it as the link preference. It establishes a new comprehensive link prediction model, which takes into account four dimensions like user attribute similarity, connection tightness of network structure (including node-based tightness and path-based tightness), asymmetric interaction intensity, and the matching degree of personality link preference. Finally, a comparative experiment is designed to verify the validity of the model experiment, which proves that the improvement of node metrics, the proposed asymmetric interactive calculation, and the calculation of personality link preferences can help improve the accuracy of link prediction to a certain extent.

Overall, this paper optimizes the personality prediction scheme for Sina Weibo. It is confirmed that there is a correlation between the user’s personality and the follow relationship. In other words, personality is an influential factor of the following relationship, which can be summarized as the link preference. It optimizes the measurement of connection tightness of network structure and adds the calculation of asymmetric interaction intensity and the matching degree of personality link preference to the social relationship prediction model, which improves the model’s accuracy. In the future, research results can be applied to various fields, including personalized recommendation (Xu et al., 2020, 2021a,b; Bao et al., 2022), sustainable tourism (Xiang et al., 2021; Wang et al., 2022), personal health (Tang Z. et al., 2021), and so on.

## Data Availability Statement

The data used to support the findings of this study are available from the corresponding author upon request. Requests to access the datasets should be directed to FB, baofuguang@126.com.

## Ethics Statement

This study was conducted according to the guidelines of the Declaration of Helsinki. The studies involving human participants were reviewed and approved by Ethics Committee of Zhejiang Gongshang University. Written informed consent to participate in this study was provided by the participants.

## Author Contributions

CJ, FB, and GL designed the study and conceived the manuscript. FB and GL implemented the simulation experiments. FB, GL, and TG drafted the manuscript. YZ, FB, and GL were involved in revising the manuscript. All authors were involved in writing the manuscript and approved its final version.

## Conflict of Interest

The authors declare that the research was conducted in the absence of any commercial or financial relationships that could be construed as a potential conflict of interest.

## Publisher’s Note

All claims expressed in this article are solely those of the authors and do not necessarily represent those of their affiliated organizations, or those of the publisher, the editors and the reviewers. Any product that may be evaluated in this article, or claim that may be made by its manufacturer, is not guaranteed or endorsed by the publisher.

## Footnotes

1
https://mp.weixin.qq.com/s/zTYKVV89ocjR2U_rnyRm1A
